# 

**Published:** 1888-03

**Authors:** 


					﻿NOTICE.
All articles for publication, books for review, business communications, etc.,
must be sent to Editors, 1123 Spruce Street, Philadelphia.
Subscribers will please notice that this Journal will be sent until arrears •
are paid and it is ordered discontinued.
Subscribers are respectfully requested to make all checks
and money-orders payable to THE IIOMG^OPATHIC
PHYSICIAN, q/nd not to either of the Editors,
				

